# Rumen microbial-driven metabolite from grazing lambs potentially regulates body fatty acid metabolism by lipid-related genes in liver

**DOI:** 10.1186/s40104-022-00823-y

**Published:** 2023-03-07

**Authors:** Zhen Li, Xingang Zhao, Luyang Jian, Bing Wang, Hailing Luo

**Affiliations:** State Key Laboratory of Animal Nutrition, College of Animal Science and Technology, China Agricultural University, Beijing, 100193 P. R. China

**Keywords:** Feeding pattern, Lamb, Lipid metabolism, Liver, Microorganism, Rumen

## Abstract

**Background:**

Lipid metabolism differs significantly between grazing and stall-feeding lambs, affecting the quality of livestock products. As two critical organs of lipid metabolism, the differences between feeding patterns on rumen and liver metabolism remain unclear. In this study, 16S rRNA, metagenomics, transcriptomics, and untargeted metabolomics were utilized to investigate the key rumen microorganisms and metabolites, as well as liver genes and metabolites associated with fatty acid metabolism under indoor feeding (F) and grazing (G).

**Results:**

Compared with grazing, indoor feeding increased ruminal propionate content. Using metagenome sequencing in combination with 16S rRNA amplicon sequencing, the results showed that the abundance of propionate-producing *Succiniclasticum* and hydrogenating bacteria Tenericutes was enriched in the F group. For rumen metabolism, grazing caused up-regulation of EPA, DHA and oleic acid and down-regulation of decanoic acid, as well as, screening for 2-ketobutyric acid as a vital differential metabolite, which was enriched in the propionate metabolism pathway. In the liver, indoor feeding increased 3-hydroxypropanoate and citric acid content, causing changes in propionate metabolism and citrate cycle, while decreasing the ETA content. Then, the liver transcriptome revealed that 11 lipid-related genes were differentially expressed in the two feeding patterns. Correlation analysis showed that the expression of *CYP4A6*, *FADS1*, *FADS2*, *ALDH6A1* and *CYP2C23* was significantly associated with the propionate metabolism process, suggesting that propionate metabolism may be an important factor mediating the hepatic lipid metabolism. Besides, the unsaturated fatty acids in muscle, rumen and liver also had a close correlation.

**Conclusions:**

Overall, our data demonstrated that rumen microbial-driven metabolite from grazing lambs potentially regulates multiple hepatic lipid-related genes, ultimately affecting body fatty acid metabolism.

**Supplementary Information:**

The online version contains supplementary material available at 10.1186/s40104-022-00823-y.

## Background

Ruminants rely on natural pastures for nutrients to grow and reproduce, with the attendant negative effects of lower growth and reduced industrial efficiency [1]. Moreover, the demand for increased husbandry output has led to a series of overgrazing problems such as the continued deterioration of grasslands. To improve the growth performance of these ruminants and alleviate the pressure of overgrazing, indoor feeding is an alternative to natural grazing [2]. However, the results of numerous studies confirmed that indoor feeding, while improving production efficiency, leads to poor flavor and fatty acids (FAs), which do not meet consumer demand for healthy green foods [3, 4]. The unique natural climatic conditions and high-quality grazing resources in Ningxia Hui Autonomous Region of China, have resulted in an excellent breed of Tan lamb with very good eating quality having a unique flavor, and relatively low saturated fatty acid (SFAs) and cholesterol content, and has appeared on the G20 Table in 2016. With the emergence of environmental issues and the quest for productivity, the feeding model of Tan lambs is gradually shifting to indoor feeding, with the consequent decline in lamb quality, especially in terms of FAs [5, 6]. The current study is thus aimed at investigating the mechanisms of differences in lipid metabolism between grazing and indoor feeding sheep.

In ruminants, the role of the rumen is crucial for the metabolism of FAs. Ruminal microorganisms synthesize odd-carbon fatty acids from propionate and valerate, as well as branched-chain fatty acids from isobutyrate, isovalerate and branched-chain amino acids, which are deposited directly or indirectly in muscle, adipose and milk [7]. Along with the synthesis of FAs, rumen microbes also break down lipids and hydrogenate unsaturated fatty acids (UFAs). Thus, both synthesis and digestion processes together have an impact on the content and composition of the final FAs flowing from the rumen [8]. A previous study assessed adipose tissue fatty acid profiles by rumen fluid, which further suggests an important association between rumen and fatty acid deposition [9]. Microbial fermentation ensures normal physiological activity, while changes in microbial community structure may lead to differences in productivity and product quality. Notably, rumen community composition is extremely sensitive to diets or feeding patterns, thereby causing changes in lipid metabolism [10, 11]. However, studies on the regulation of fatty acid metabolism by rumen microbes in sheep are scarce and need to be further developed. The short-chain fatty acids produced in the rumen are absorbed through the rumen wall, while the rest of the lipid digestion products are absorbed in the posterior part of the small intestine and then enter the peripheral circulation to reach the liver and other tissues. As the ultimate distributor of nutrients for the growth of peripheral tissues and organs, the liver is also the central organ of lipid metabolism in the body [12]. The hepatic lipid machinery is complex and highly coordinated, and is extremely susceptible to the effects of diet, environment and other factors [13, 14].

Based on the sensitivity of microbial community composition and liver biology to diets or feeding methods, it is valuable to investigate the influence of rumen microorganisms and liver metabolism by changing feeding patterns to regulate the FAs metabolism of the organism. To the best of our knowledge, there are few researches on variations in rumen and liver metabolism between grazing and indoor feeding lambs. The relationship between rumen microorganisms and FAs metabolism in sheep is also lacking. Thus, this study investigated the changes in specific microorganisms in the rumen ecosystem and liver lipid metabolism under grazing and indoor feeding practices and their potential effects on fatty acid deposition in lamb from a multi-omics perspective.

## Materials and methods

### Grassland preparation

The experiment was conducted in the desert and semi-desert steppes of Dashuikeng, Yanchi County, Ningxia, China (106°58′E, 37°26′N; elevation 1400 m). The area's average annual temperature was 8.3 °C, and the average yearly precipitation was 282.3 mm, with most of it falling between June and September. The experimental pasture was about 1900 m long from east to west and 250 m wide from north to south, and divided into eight grazing plots with the same area. Grazing was carried out in one plot each day and rotated in the eight plots. The vegetation composition of the eight plots was basically the same.

### Experimental design and sampling

Twenty-six male Tan lambs (*Ovis aries*) from the same flock, approximately 120 days of age with similar body weight (25.06 ± 0.32 kg) were selected and randomly divided into one of the two feeding systems (*n* = 13 per group): the indoor feeding group (F; feed twice a day at 8:00 and 17:00) and the natural pasture grazing group (G; graze from 7:00 to 19:00). Lambs in the F group were fed pellets supplemented with hay separately (Table S1) in individual pens (size: 1.5 m × 3 m). The experiment lasted for 83 d, including a 10-d adaptation period. During the adaptation period, the daily feeding amount was adjusted based on the actual intake of the previous day to ensure a 15% surplus. Clean water was available for the animals all the time. Serum samples were collected from the jugular vein through a 10-mL vacuum tube in the morning before feeding on the last day of the experiment. The serum samples were separated by centrifugation of blood and stored at −20 °C for the determination of serum variables. At the end of the feeding experiment, Tan lambs were fasted for 24 h and prevented from drinking for 12 h before slaughter. The liver and rumen samples were collected after slaughter and stored in liquid nitrogen for subsequent analysis.

### Analysis of muscle and herbage fatty acid composition

After intramuscular fat was extracted from *longissimus dorsi* (LD) muscle and herbage by using a chloroform/methanol mixture, FAs in intramuscular fat were quantified using an Agilent 6890 gas chromatograph coupled with a mass spectrometer (GC/MS, Agilent Technologies Inc., Santa Clara, CA, USA). More details can be found in Guo’s research [6]. We selected the 16 lambs that underwent rumen and liver metabolism measurements, and then obtained the FAs content in their LD muscle, which are as follows: C18:2n6 (2.517 ± 0.147), C18:3n3 (0.217 ± 0.022), C20:3n6 (0.081 ± 0.005), C20:4n6 (1.054 ± 0.069), C20:5n3 (0.078 ± 0.009), C22:6n3 (0.039 ± 0.005), total FA (29.818 ± 1.654), n-3 polyunsaturated fatty acids (PUFAs) (0.334 ± 0.035), n-6 PUFAs (3.652 ± 0.208), n-6/n-3 PUFAs (12.889 ± 1.326). FA content was expressed as an mg/100 g of fresh meat. Based on the fatty acid content in the herbage and the estimated forage intake, we calculated the daily fatty acid intake of grazing sheep as follows: C12:0 (50.14 mg), C14:0 (99.48 mg), C16:0 (1589.48 mg), C16:1 (51.64 mg), C17:0 (33.36 mg), C18:0 (277.71 mg), C18:1n9c (1160.59 mg), C18:2n6 (4331.75 mg), C18:3n3 (1248.74 mg), C20:0 (196.46 mg), C20:1 (28.61 mg), C21:0 (98.35 mg), C20:2 (28.57 mg), C22:0 (292.76 mg), C22:1n9 (26.62 mg), C23:0 (119.74 mg), C24:0 (212.77 mg), total FAs (9846.78 mg).

### Rumen fermentation characteristics

After opening the sheep's abdominal cavity, the internal organs were immediately dissected and the rumen was separated. Rumen fluid samples were collected by straining the ruminal content through a four-layer gauze. The pH value of rumen fluid was immediately measured using an electric pH meter (PHS-3C, Shanghai Leijun Experimental Instrument Co., Ltd., Shanghai, China). Then rumen fluid samples were stored in liquid nitrogen for subsequent analysis. The content of volatile fatty acid (VFA) in rumen fluid was determined by using the Trace 1300 gas chromatograph model (Thermo Fisher Scientific, Waltham, MA, USA). The content of ammonia nitrogen was determined by phenol-sodium hypochlorite colorimetry [15].

### Serum and liver biochemical parameters

Serum and liver tissue samples were stored at −20 ℃. We added 1 g of liver into 9 mL normal saline, and then ground into 10% liver tissue homogenate. The total antioxidant capacity (T-AOC), low-density lipoprotein (LDL), high-density lipoprotein (HDL), blood urea nitrogen (BUN), triglyceride, cholesterol, nonestesterified fatty acid (NEFA), fatty acid synthase (FAS) and acetyl-CoA carboxylase (ACC) in the serum and liver were measured using the corresponding colorimetric assay kit (Nanjing Jiancheng Bioengineering Institute, Nanjing, China).

### Ruminal 16S rRNA sequencing

Genomic DNA was extracted from rumen content using HiPure Stool DNA Kits (Magen, Guangzhou, China). The V3 + V4 region of 16S rRNA was amplified with specific primers with barcode: 341F: 5'-CCTACGGGNGGCWGCAG-3' and 806R: 5'-GGACTACHVGGGTATCTAAT-3'. The PCR products after amplification were purified using AMPure XP Beads. Then, ABI StepOnePlus^TM^ Real-Time PCR System (Applied Biosystems, Foster City, CA, USA) was used for quantification. Machine pooling and sequencing were performed according to the PE250 mode of Novaseq 6000. According to the standard protocol, purified amplicons were collected on the Illumina platform at equal mole-concentration and sequenced in pairs. The raw reads obtained by sequencing were filtered and corrected by DADA2, and the non-redundant reads and their corresponding abundance information were output. Then the reads were spliced into tags, and the chimeric tags were removed to obtain the Tag sequence and abundance information for subsequent analysis, namely ASV sequence and ASV abundance information. The representative ASV sequences were classified into organisms by a naive Bayesian model using RDP classifier (version 2.2) based on the SILVA database (version 132), with a confidence threshold value of 0.8 [16]. Based on the ASV sequence and abundance data, species annotation, species composition analysis and community function prediction were carried out, and we compared the differences between the two groups. Good’s coverage, Chao1, Simpson, and other alpha indexes were calculated in QIIME (version 1.9.1) and statistical analysis of Anosim (analysis of similarities) test and Welch’s *t*-test were calculated in R project Vegan package (version 2.5.3).

The raw reads of 16S rRNA sequence were deposited into the NCBI Sequence Read Archive (SRA) database (project number, PRJNA859697, accession number, SRP386848).

### Ruminal metagenomics analysis and data processing

Genomic DNA was extracted using HiPure Bacterial DNA Kits (Magen, Guangzhou, China) according to the manufacturer’s instructions and subsequently DNA quality was tested. Qualified genomic DNA was firstly fragmented by sonication to a size of 350 bp, and then end-repaired, A-tailed, and adaptor ligated using NEBNext® ULtra™ DNA Library Prep Kit for Illumina (New England BioLabs, Ipswich, MA, USA) according to the preparation protocol. DNA fragments with a length of 300–400 bp were enriched by PCR. At last, PCR products were purified using the AMPure XP system (Beckman Coulter, Brea, CA, USA) and libraries were analyzed for size distribution by 2100 Bioanalyzer (Agilent, Santa Clara, CA, USA) and quantified using real-time PCR. Genome sequencing was performed on the Illumina Novaseq 6000 sequencer using pair-end technology (PE 150). The raw data from the Illumina platform was filtered using FASTP (version 0.18.0) according to the following criteria: 1) removal of reads with ≥ 10% unidentified nucleotides (N); 2) removal of reads with ≥ 50% bases having phred quality scores ≤ 20; and 3) removal of reads aligned to barcode adapters [17]. After filtering, the resulting clean reads were used for genome assembly. Clean reads of each sample were assembled individually using MEGAHIT (version 1.1.2) to generate sample-derived assembly. We used MetaGeneMark (version 3.38) to predict genes based on the final assembly contigs. All predicted genes of length > 300 bp were merged according to 95% identity and 90% coverage of reads using CD-HIT (version 4.6) to reduce the number of redundant genes in downstream assembly steps. Using Bowtie (version 2.2.5) to count reads numbers, the reads were realigned to the predicted genes [18]. The final gene catalog was obtained from non-redundant genes with gene reads count greater than 2.

Several complementary methods were used to annotate the assembled sequences. The unigenes were annotated using DIAMOND (version 0.9.24) by aligning with the deposited ones in the database of Kyoto Encyclopedia of Genes and Genomes (KEGG). Additional annotation was carried out based on the Carbohydrate-Active enZYmes (CAZy). Statistical analysis of Welch’s *t*-test was calculated using R project Vegan package. Biomarker features in each group were screened by LEfSe software (version 1.0).

The raw reads of rumen metagenome sequences were deposited into the NCBI SRA database (project number, PRJNA860332, and accession number, SRP387213).

### Metabolome analysis in rumen fluid and liver tissue

The rumen fluid and liver samples were lyophilized, dissolved in methanol solution (−20 °C), and vortexed for 1 min. Centrifuged at 12,000 r/min for 10 min at 4 °C, and 450 μL of supernatant was taken for vacuum concentration. The samples were dissolved in 150 μL 2-chlorobenzalanine (4 μL/L), and the supernatant was filtered through a 0.22 µm membrane to obtain the prepared samples for Liquid Chromatography-Mass Spectrometry (LC–MS). Chromatographic separation was accomplished in a Thermo Ultimate 3000 system equipped with an Acquity UPLC® HSS T3 (150 mm × 2.1 mm, 1.8 µm, Waters, Milford, MA, USA) column maintained at 40 ℃. The temperature of the autosampler was 8 ℃. Gradient elution of analytes was carried out with 0.1% formic acid in water and 0.1% formic acid in acetonitrile (positive model) or 5 mmol/L ammonium formate in water and acetonitrile (negative model) at a flow rate of 0.25 mL/min. Injection of 2 μL of each sample was done for gradient elution after equilibration. Electrospray ionization positive-ion and negative-ion modes were used for detection. The experiments were executed on the Thermo Q Exactive mass spectrometer with a spray voltage of 3.5 kV (positive model) and 2.5 kV (negative model) with the 325 ℃ of capillary temperature. Sheath gas and auxiliary gas were set at 30 and 10 arbitrary units, respectively. The analyzer scanned over a mass range of *m/z* 81–1000 for full scan at a mass resolution of 70,000. Proteowizard (version 3.0.8789) was used to transform the raw data files into mzXML format. Peak identification, peak filtering, and peak alignment for each metabolite were performed using the R (version 3.3.2) package XCMS [19]. The following were the major parameters: bw = 5, quality deviation = 15, peakwidth = c (5, 30), mzwid = 0.01, mzdiff = 0.01, method = "centWave". For further examination, the mass-to-charge ratio (*m/z*), retention duration and intensity, and positive and negative precursor molecules were employed. Batch normalization was used to convert peak intensities to overall spectral intensity. The precise molecular formula (molecular formula error < 20) was used to identify metabolites. To validate metabolite annotations, peaks were matched using Metlin (http://metlin.scripps.edu) and MoNA (https://mona.fiehnlab.ucdavis.edu).

To extract the most useful information, the collected multidimensional data were reduced and classified, including unsupervised principal component analysis (PCA) and discriminant analysis of squares (PLS-DA) with minimal supervision. The first principal component of the variable importance in the projection (VIP) was obtained from PLS-DA to refine this analysis. Metabolites with a VIP value exceeding 1 were further applied to *t*-test at the univariate level to measure the significance of metabolite in two groups, and the *P* value less than 0.05 was deemed as statistically significant. Receiver operating characteristic (ROC) curve analysis by R pROC package to evaluate the predictive power of each of the discriminant metabolites. The area under the curve (AUC) was computed via numerical integration of the ROC curves. The metabolite signature that has the largest AUC was identified as having the strongest predictive power for discriminating the two groups. The fold-change value of each metabolite was calculated by comparing the mean value between G and F. The differential metabolites (DFMs) were further identified and validated by KEGG. The KEGG database was applied to the enrichment analysis of the KEGG metabolic pathway based on the DFMs. The calculated *P*-value was gone through false discovery rate (FDR) correction, taking FDR ≤ 0.05 as a threshold. Pathways that satisfied this condition were defined as significantly enriched in DFMs.

### Transcriptome sequencing and quantitative real-time PCR validation of liver

Total RNA was extracted according to the manufacturer’s protocol using a Trizol reagent kit (Invitrogen, Carlsbad, CA, USA). RNase-free agarose gel electrophoresis was used to verify RNA quality using an Agilent 2100 Bioanalyzer (Agilent Technologies, Palo Alto, CA, USA). Following total RNA extraction, eukaryotic mRNA was isolated using Oligo (dT) beads, whereas prokaryotic mRNA was enriched using the Ribo-Zero^TM^ Magnetic Kit (Epicentre, Madison, WI, USA) to remove rRNA. The enriched mRNA was then fragmented into small fragments with fragmentation buffer before being reverse transcribed into cDNA with random primers. DNA polymerase I, RNase H, dNTP, and buffer were used to make second-strand cDNA. The cDNA fragments were then purified using a QiaQuick PCR extraction kit (Qiagen, Venlo, The Netherlands), end repaired, poly (A) added, and ligated to the Illumina sequencing platform. The ligation products were sized by using agarose gel electrophoresis, then PCR amplified and sequenced on an Illumina HiSeq2500.

To obtain high-quality clean reads, raw reads obtained from the sequencing machines were further filtered by fastp (version 0.18.0). Reads were mapped to the ribosome RNA (rRNA) database by using the short reads alignment tool Bowtie2 (version 2.2.8) to eliminate the rRNA mapped reads [18]. The remaining clean reads were further used in assembly and gene abundance calculation. An index of the reference genome was built, and paired-end clean reads were mapped to the reference genome using HISAT2. 2.4. For each transcriptional region, FPKM values (fragment per kilobase of transcript per million mapped reads) were calculated using StringTie (version 1.3.1) software to quantify expression abundance and variation [20].

RNA differential expression analysis was performed by DESeq2 software between two groups [21]. The transcripts with the parameter of FDR below 0.05 and absolute fold change ≥ 2 were considered as differentially expressed transcripts. Differential expression genes (DEGs) in two groups were functionally annotated by gene ontology (GO) enrichment analysis. Physiological metabolism events and signal pathways of the DEGs were assessed using KOBAS software to test the statistical enrichments of the DEGs in KEGG pathways. The calculated *P*-value was gone through FDR correction, taking FDR ≤ 0.05 as a threshold. Pathways of GO and KEGG analysis meeting this condition were defined as significantly enriched pathways in DEGs. After selecting the eleven genes that were enriched in the lipid-related pathway, we conducted quantitative real-time PCR (qPCR) to validate the expression.

The raw reads of transcriptome sequences were deposited into the NCBI SRA database (project number, PRJNA859628 and accession number, SRP386837).

### Statistical analyses

The rumen fermentation characteristics, serum and liver biochemical parameters were compared using *t*-test by IBM SPSS Statistics for Windows (version 22.0, IBM Corp, Armonk, NY, USA). Statistical significance was defined at *P* < 0.05. Correlation analyses were conducted by Pearson correlation analysis. Statistical significance was defined at *P* < 0.05.

## Results

### Ruminal fermentation parameters

**Fig. 1:**
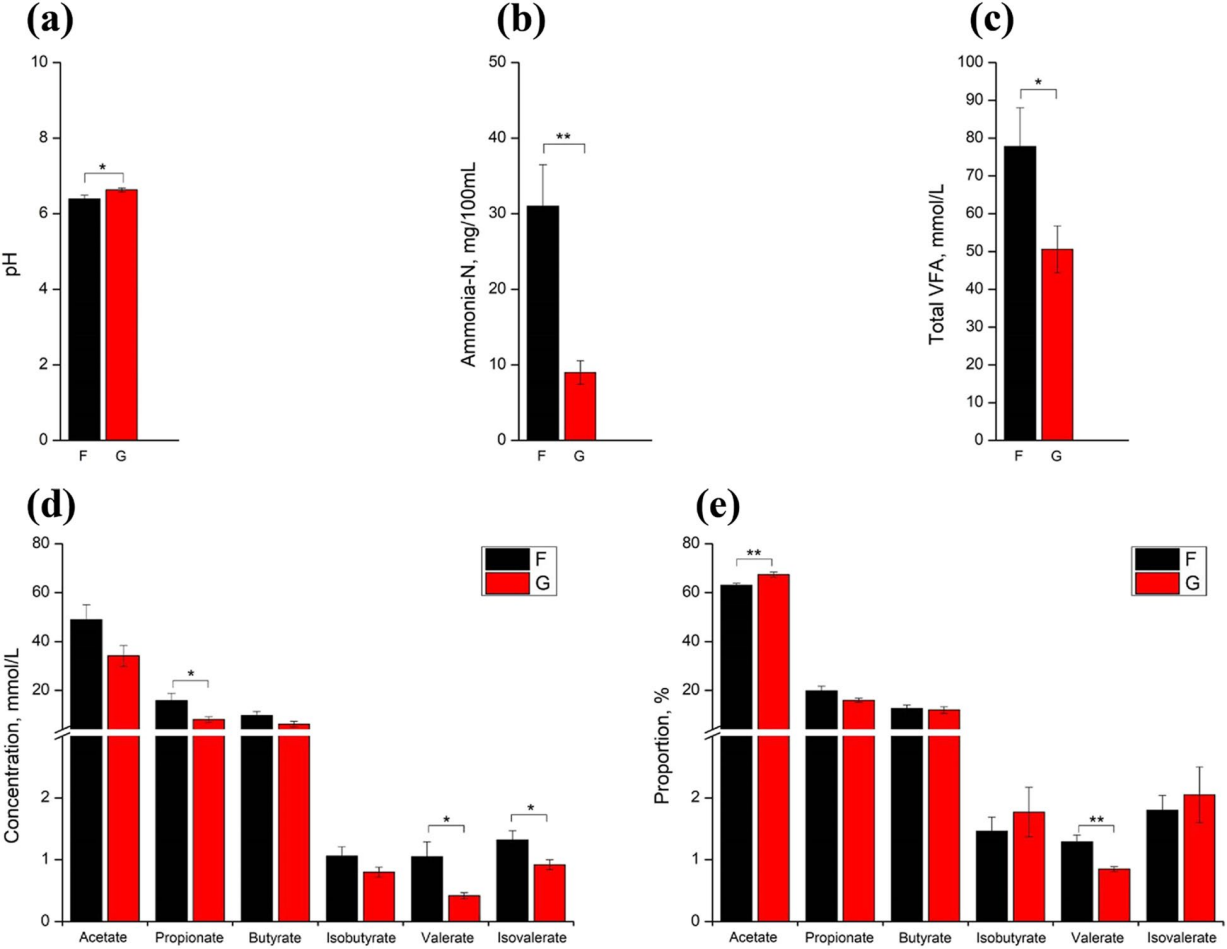
Effects of feeding patterns on the rumen fermentation parameters: including rumen pH (**a**), ammonia-N (**b**) and the concentration of total VFA (**c**). Comparisons of the concentrations (**d**) and proportion (**e**) of VFA in the rumen between the F and G groups (*n* = 6 per group). ^*^P < 0.05; ^**^P < 0.01. “F” means indoor feeding group; “G” means grazing group; the bar in each column means standard error. VFA, volatile fatty acid

Compared with the F group, grazing enhanced the pH of rumen fluid (*P* < 0.05, Fig. 1a). The ammonia-N concentration was greater in the stall-feeding lambs than in the G groups (*P* < 0.01, Fig. 1b). As for the fermentation indicators (Fig. 1c–e), the total VFA, propionate, valerate, isovalerate, and valerate proportion were increased in stall feeding sheep, while acetate proportion was decreased (*P* < 0.05).

### Serum and liver biochemical parameters

**Fig. 2:**
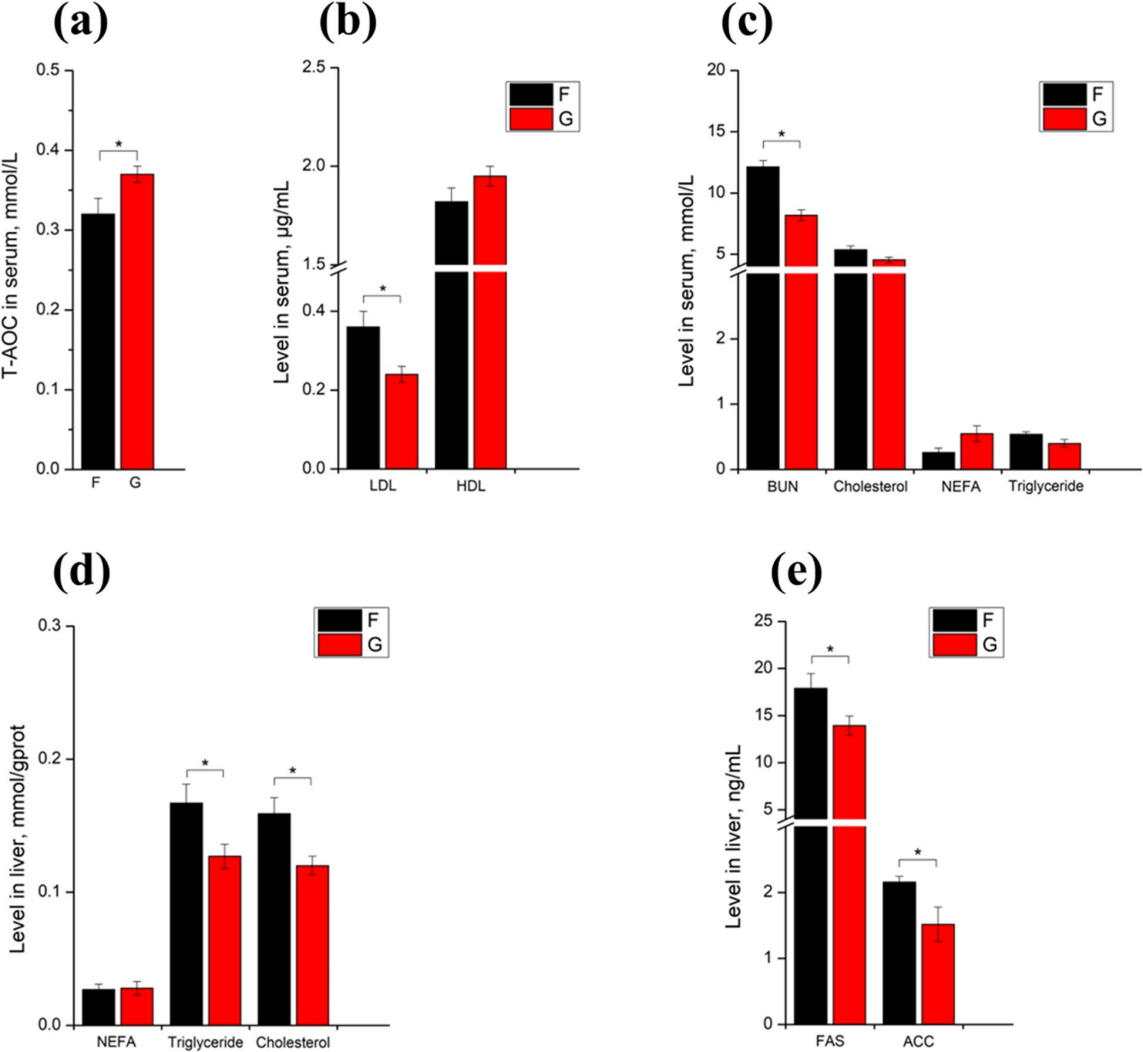
Comparisons of the biochemical parameters in the serum (**a**–**c**) and liver (**d**–**e**) between the F and G groups. ^*^P < 0.05; ^**^P < 0.01. “F” means indoor feeding group; “G” means grazing group; the bar in each column means standard error. T-AOC, total antioxidant capacity. LDL, low-density lipoprotein. HDL, high-density lipoprotein. BUN, blood urea nitrogen. NEFA, nonestesterified fatty acid. FAS, fatty acid synthase. ACC, acetyl-CoA carboxylase

In the biochemical parameters of serum (Fig. 2a–c), the contents of LDL and BUN were higher in the F group (*P* < 0.05), whereas T-AOC was lower in the F group (*P* < 0.05). In the liver (Fig. 2d–e), the grazing pattern significantly reduced the contents of triglyceride, cholesterol, FAS, and ACC (*P* < 0.05).

### Ruminal microbial community characteristics

**Fig. 3:**
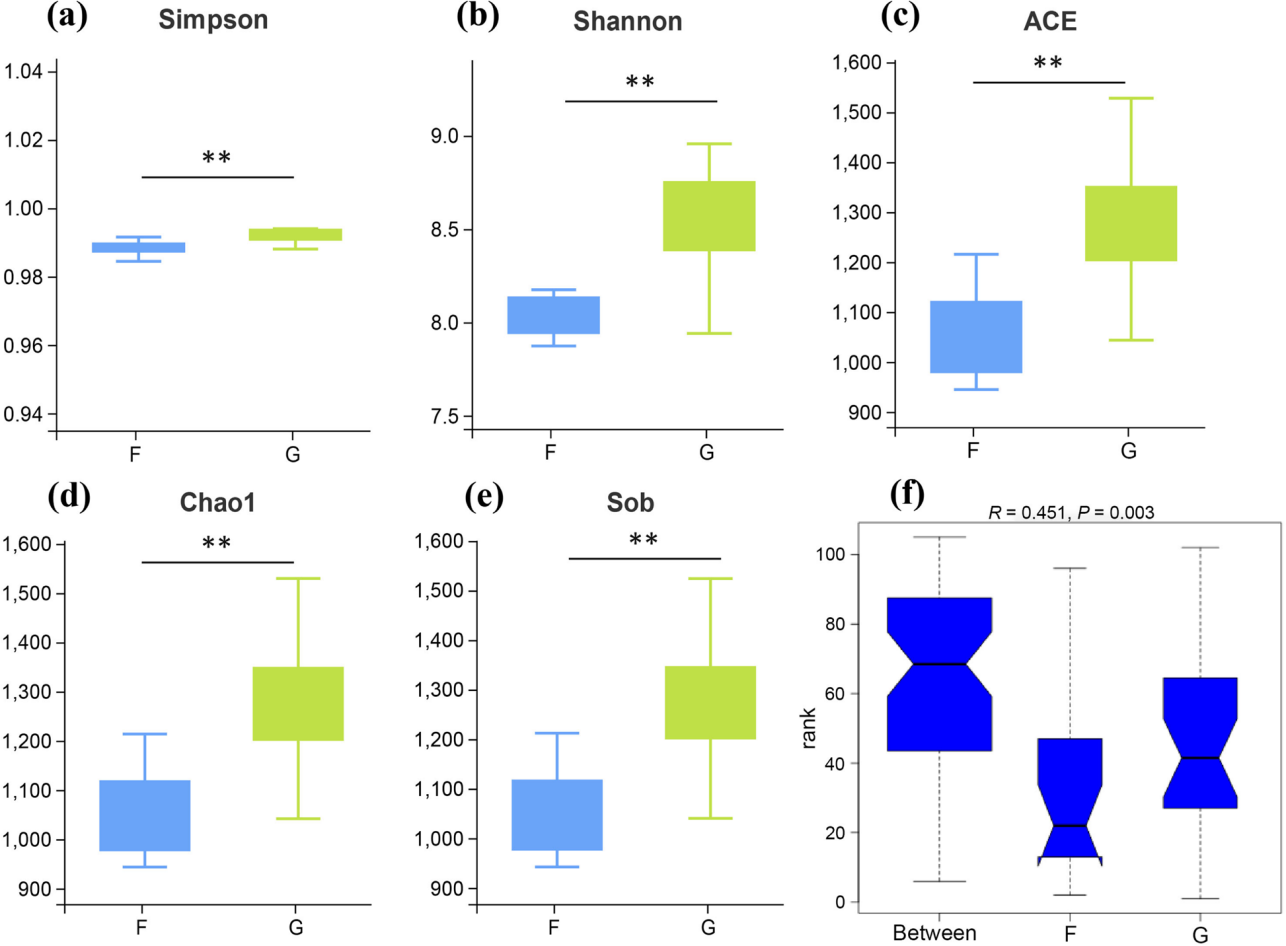
Analysis of rumen microbial diversity of sheep in the F and G groups by 16S rRNA sequencing. Alpha diversity analysis (**a**–**e**). Anosim (analysis of similarities) (**f**) based on Bray–Curtis distances between the F (*n* = 7) and G (*n* = 8) group. ^*^P < 0.05, ^**^P < 0.01. “F” means indoor feeding group; “G” means grazing group

**Fig. 4:**
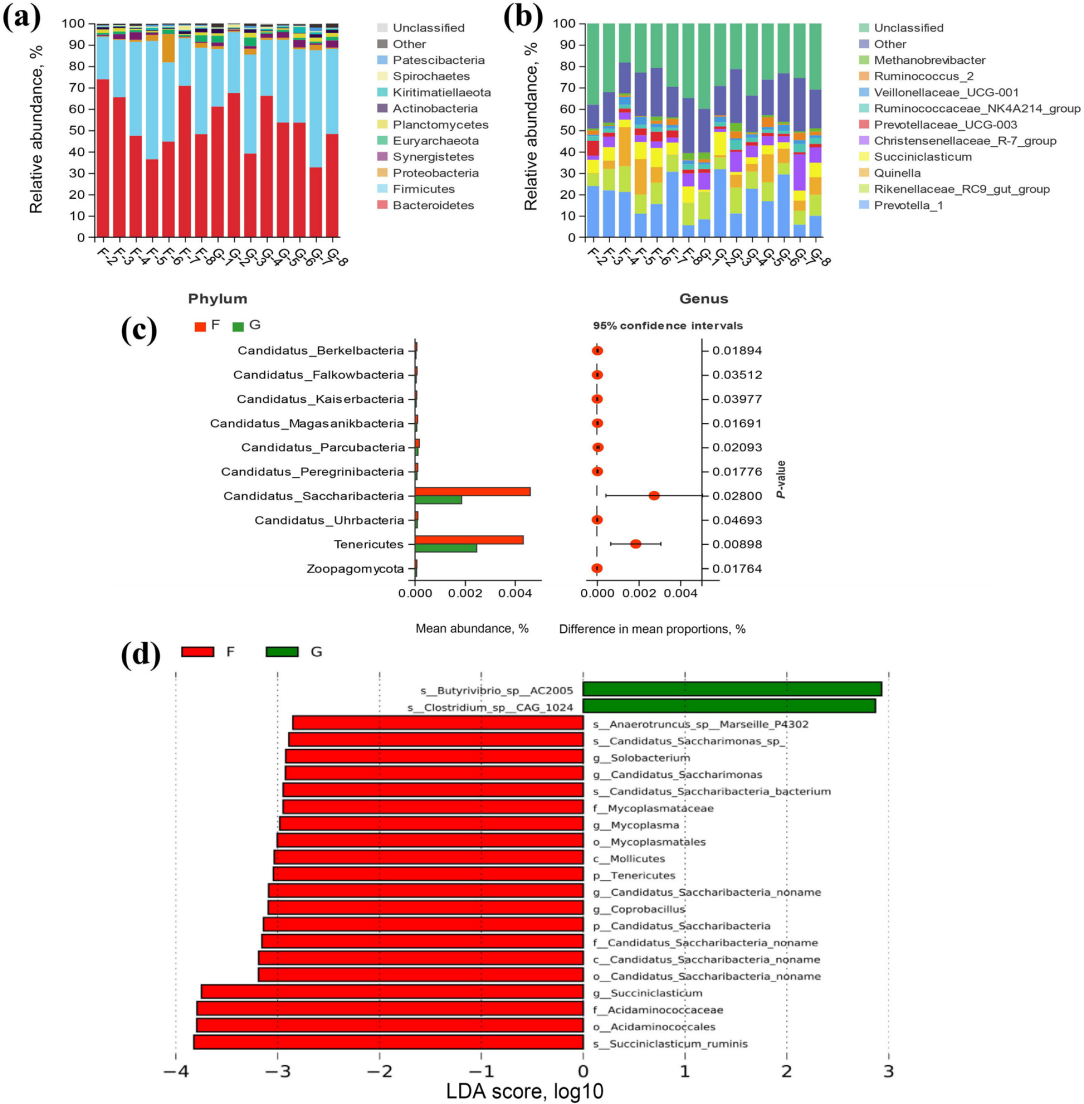
Relative abundance of bacterial community proportions at the phylum (**a**) and genus (**b**) levels are compared between the two groups based on the 16S rRNA data (as a percentage of total sequences). The most ten abundant differential phyla screened by metagenomic sequencing based on the Welch's *t*-test (*n* = 4 per group) (**c**). The significantly differential microorganisms based on the linear discriminant analysis effect size (LEfSe) cladogram in metagenomic sequencing (**d**), and the differences are represented by the color of the group. “F” means indoor feeding group; “G” means grazing group

There was an average of 103,855 ± 1195 clean reads (mean ± standard error of the mean [SEM]) per sample via 16S rRNA sequencing. The Good’s coverage of all samples was greater than 0.99, indicating that the sequencing data of 16S rRNA was sufficient (Table S2). As shown in Fig. 3, the Shannon, Simpson, Ace, Chao1 and Sob indexes of bacterial richness and diversity were significantly higher in G group than F group (*P* < 0.01). The Anosim based on Bray–Curtis distances showed significant differences between the two groups (Fig. 3, *P* < 0.01). At the phylum level (Fig. 4a), most sequences were assigned to Bacteroidetes (53.90% ± 3.25%) and Firmicutes (36.09% ± 2.76%). At the genus level (Fig. 4b), the most predominant genus was *Prevotella_1* (17.68% ± 2.24%) in the rumen. Based on the bacterial diversity and Bray–Curtis metric, eight samples (4 lambs per group) were selected and used for shotgun metagenome sequencing. Metagenome sequencing generated 69.17 ± 0.72 million raw reads. After quality control and removing host genes, 68.93 ± 0.72 million clean reads were retained. After assembly, a total of 1,361,185 and 1,576,316 contigs were generated in F and G groups, respectively. We then performed gene prediction and clustering for contigs larger than 500 bp and obtained 432,423.75 ± 29,037.86 non-redundant genes. Through the sequencing, there were four specific phyla in the G group, which were Euryarchaeota, Nanoarchaeota, Thaumarchaeota and Abditibacteriota (Fig. S1a). Based on the Welch’s *t*-test, with *P*-value less than 0.05 as the threshold, a total of 35 differential phyla were screened, all of which showed higher abundance in the F group, including Tenericutes and Candidatus_Saccharibacteria (*P* < 0.05, Fig. 4c). Linear discriminant analysis effect size (LEfSe) was used to screen the main specific microorganisms between the two groups, and we found that the abundance of *Butyrivibrio_sp_AC2005* and *Clostridium_sp_CAG_1024* in the G group was higher than that in the F group. However, the abundance of *Succiniclasticum_ruminis*, Acidaminococcales, Acidaminacoccaceae, *Succiniclasticum, Coprobacillus* and *Candidatus_Saccharimonas* showed opposite changes (LDA > 2.8, Fig. 4d).

### Functions of the rumen microbiome

**Fig. 5:**
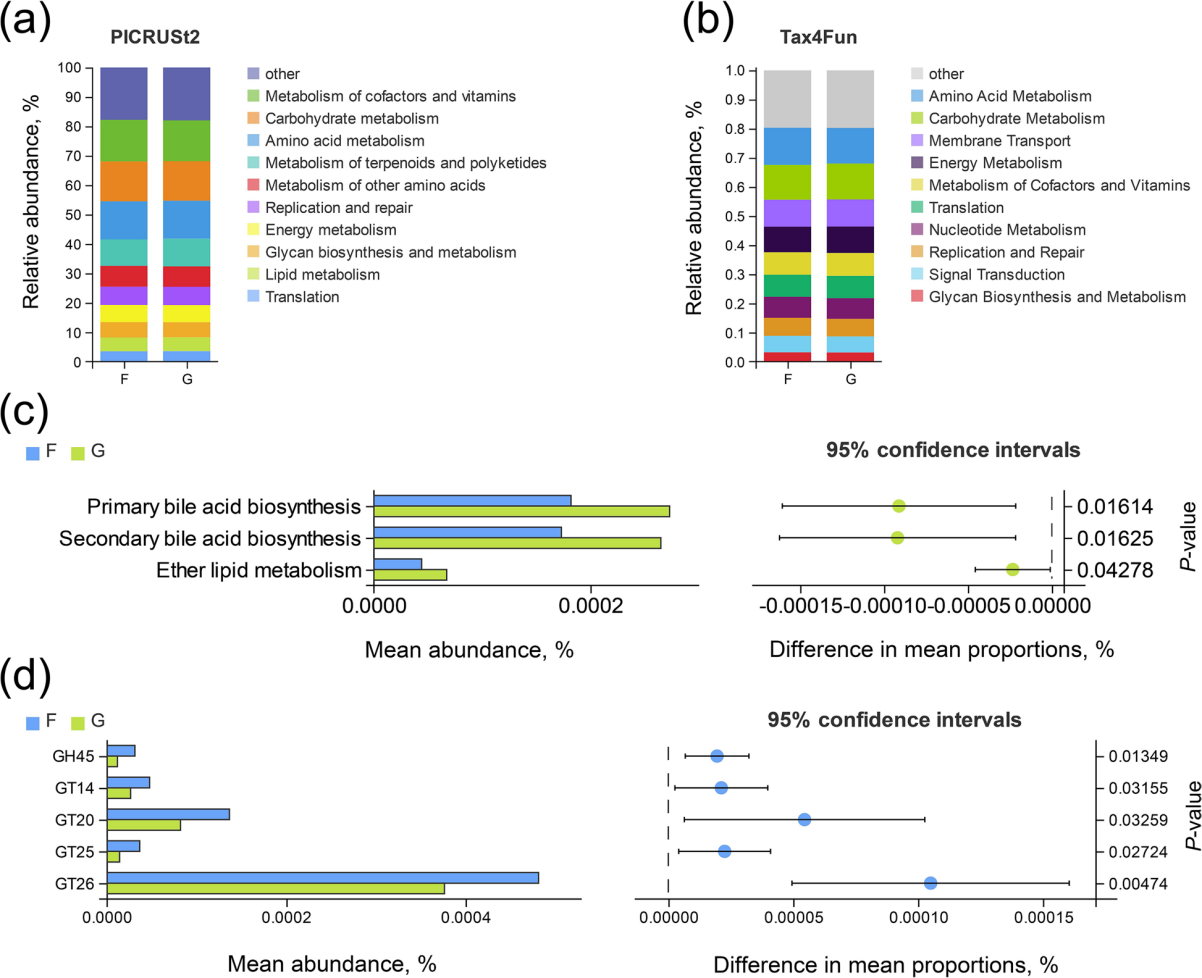
Functional prediction of the rumen microbiota based on PICRUSt2 (**a**) and Tax4Fun (**b**) is performed on 16S rDNA data. Prediction of microbial functional differences based on Tax4Fun by the Welch's *t*-test in 16S rDNA sequencing (**c**). Comparisons of the abundance of CAZymes genes of rumen microbiomes in the F and G groups by the Welch's *t*-test in metagenomic sequencing (**d**). “F” means indoor feeding group; “G” means grazing group

The Tax4Fun- and PICRUSt2-based functional predictions revealed two important functions of the rumen microbiota (Fig. 5a–b). These functions were “carbohydrate metabolism” and “amino acid metabolism”. Based on Tax4Fun predictions of microbial functional differences, three lipid metabolism pathways were screened, including primary bile acid synthesis, secondary bile acid synthesis, and etheric lipid metabolism, all of which were significantly higher in the grazing group than stall-feeding group (Fig. 5c). Through metagenomic sequencing, among the unique genes derived from the rumen microflora, 71.92% genes were classified into KEGG pathways, and 11.96% genes were classified into CAZymes. Based on the KEGG database for functional annotation of metagenome data, both groups shared 118 pathways. According to Welch's *t*-test (*P* < 0.05), two pathways, ascorbate and aldarate metabolism and penicillin and cephalosporin biosynthesis, were significantly different between the two groups (Fig. S1b). CAZy function can be used to explore the contribution of microorganisms to carbohydrate metabolism, and we found the highest percentage of two major classes of glycoside hydrolases (GH) and glycosyltransferases (GTs) at level A. At level B, based on Welch's *t*-test, the following differential enzyme families: GH45, GT14, GT20, GT25 and GT26 were found to be higher in the stall-feeding group than in the G group (Fig. 5d). Also, the reporter score analysis showed that three pathways, namely beta-alanine metabolism, alpha-linolenic acid metabolism, and biosynthesis of unsaturated fatty acid, were significantly different between the two groups (Fig. S1c). To explore the potential microbial functions, Pearson correlations were constructed between the microorganisms and the VFA in the rumen. As shown in Table S3, the abundance of *Succiniclasticum* and Acidaminococcales was significantly and positively correlated with propionate and total VFA concentrations. Also, the abundance of *Candidatus-Saccharimonas* showed a positive correlation with the concentration of valerate and total VFA.

### Rumen and liver metabolome

**Fig. 6:**
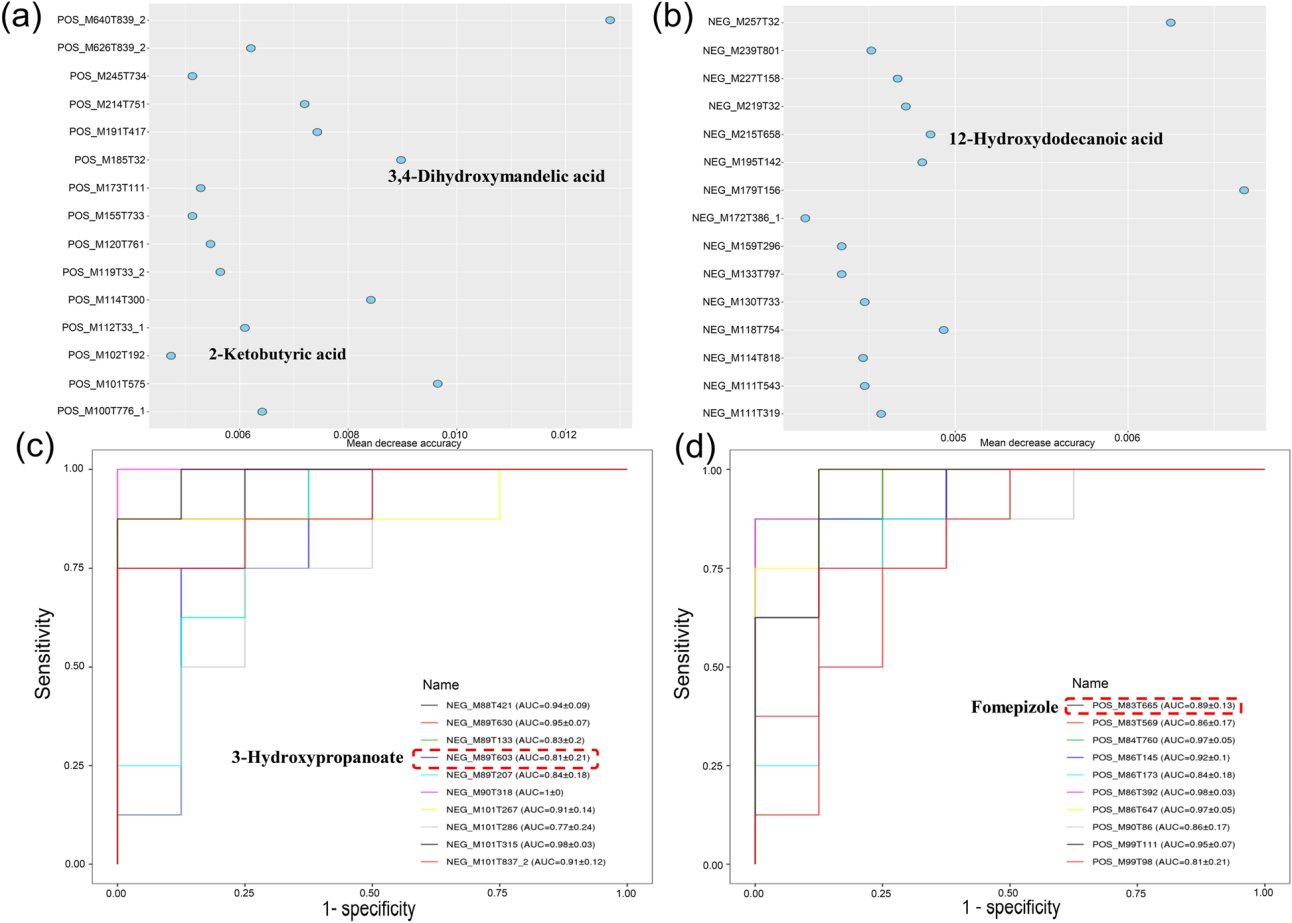
The top 15 high-contribution differential metabolites in the rumen are screened out by random forest analysis in the positive (**a**) and negative (**b**) ion mode. The top 10 high-contribution differential metabolites in the liver are screened out by AUC area calculation in the positive (**c**) and negative (**d**) ion mode. The annotated metabolites are named in the figure (*n* = 8 per group)

In the rumen metabolome, 20,058 positive ion peaks and 13,031 negative ion peaks were detected by positive ion mode and negative ion mode detection, respectively. Good separation of the rumen metabolites among the two groups was achieved in the PLS-DA score plots of the negative ion mode (Fig. S2). By *t*-test and VIP filtering of relative concentrations of rumen metabolites, 31 DFMs were identified between the two groups in the positive ionization mode; 15 of them were up-regulated and 16 were down-regulated; 52 differential peaks were identified between the two groups in the negative ionization mode, 27 of them were up-regulated and 25 were down-regulated. After finding the metabolites, the pathway enrichment analysis by KEGG was performed for the DFMs. As shown in Fig. S3a, the top 20 enriched pathways related to lipid metabolism included biosynthesis of unsaturated fatty acids, propanoate metabolism and fatty acid biosynthesis, among which biosynthesis of unsaturated fatty acids was the significantly enriched pathway. Among the DFMs enriched in this pathway, UFAs including icosapentaenoic acid (EPA), docosahexaenoic acid (DHA) and oleic acid were shown to be upregulated in the G group. And the SFAs decanoic acid showed down-regulation in the fatty acid biosynthesis pathway. By random forest analysis, the top 15 items screened out by Mean Decrease Accuracy could be used as high contributing DFMs with annotated names including 3,4-dihydroxymandelic acid, 2-ketobutyric acid and 12-hydroxydodecanoic acid (Fig. 6a). And we noted that 2-ketobutyric acid was enriched in the propionate metabolism pathway.

For liver metabolism, 7125 positive ion peaks and 4629 negative ion peaks were detected by positive and negative ion mode detection, respectively. Good separation of the rumen metabolites among the two groups was achieved in the PLS-DA score plots of the positive and negative ion modes (Fig. S2). By *t*-test and VIP filtering of relative concentrations of rumen metabolites, 430 DFMs were identified between the two groups in the positive ionization mode; 225 of them were up-regulated and 205 were down-regulated; 199 differential peaks were identified between the two groups in the negative ionization mode, 99 of them were up-regulated and 100 were down-regulated. The KEGG pathway enrichment analysis shown in Fig. S3b revealed the top 20 pathways that were affected by each of the two different feed regimes. Among them, the pathways related to lipid metabolism include primary bile acid biosynthesis, glycerophospholipid metabolism, and sphingolipid metabolism. Moreover, we observed that icosatrienoic acid (ETA), which was rich in unsaturated fatty acids metabolism pathway, was upregulated in the G group. Subsequently, we plotted ROC curves and calculated AUC areas. The top 10 AUC areas were screened for annotated DFMs, including fomepizole and 3-hydroxypropanoate, with 3-hydroxypropanoate being down-regulated in the G group and also enriched in the propionate metabolism pathway (Fig. 6b). Finally, citric acid enriched in the citric acid metabolic pathway was also found to be downregulated in the grazing lambs.

### Liver transcriptome analysis

**Fig. 7:**
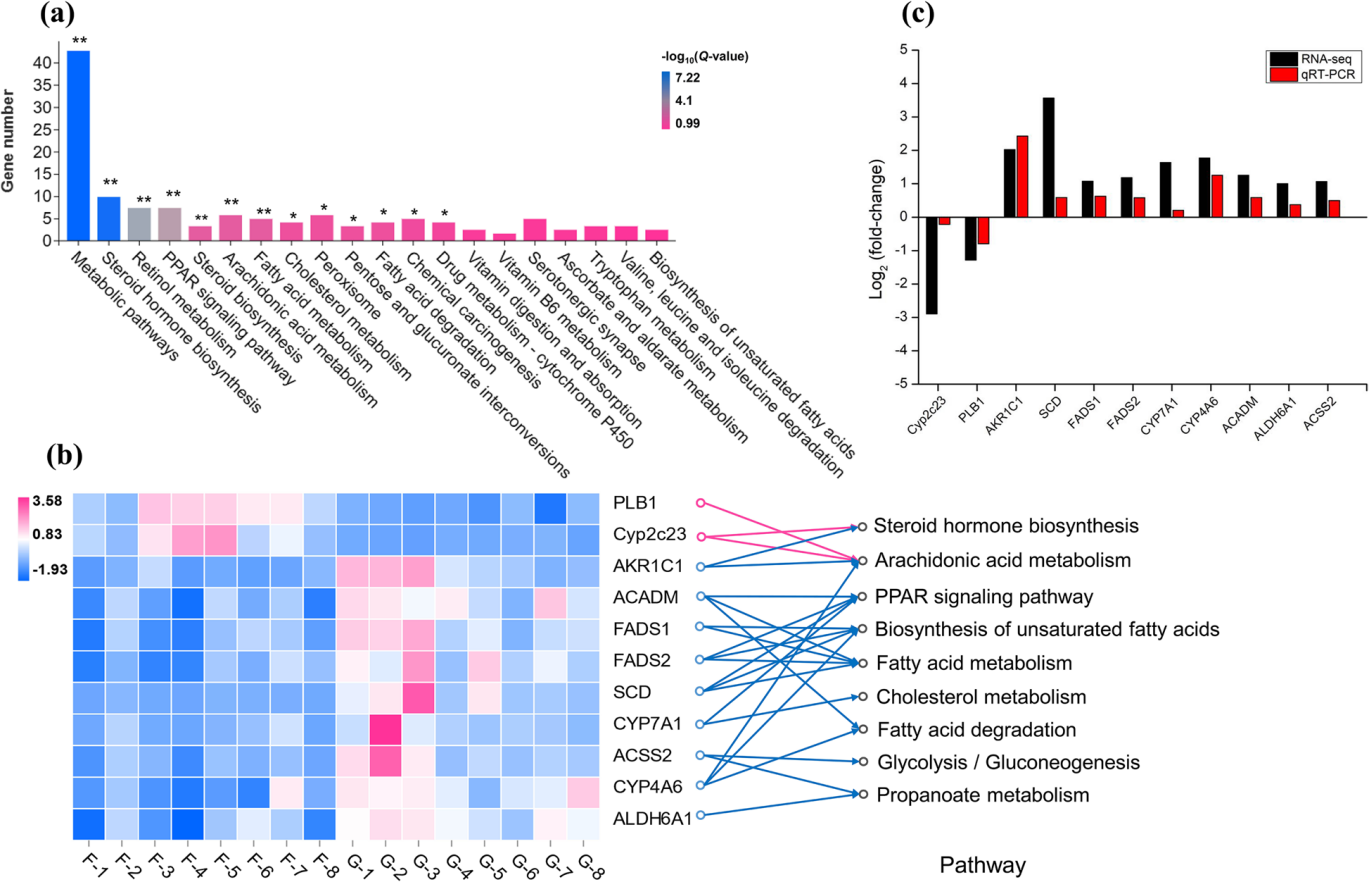
The top 20 enriched pathways of differential expression genes (DEGs) between the F and G groups lambs’ liver by KEGG analysis (**a**). The ordinate is the number of DEGs enriched into the pathway; *Q-value < 0.05; **Q-value < 0.01. Expression of differential genes involved in lipid metabolism in the F and G groups (**b**). Heatmap colors indicate FPKM values. The arrow represents the gene enrichment pathway, in which the pink arrow represents the down-regulated genes and the blue arrow represents the up-regulated genes. The verification of candidate genes expression in RNA-seq (*n* = 8 per group) by quantitative real-time PCR test (*n* = 4 per group) (**c**). “F” means indoor feeding group; “G” means grazing group

To investigate the differences in the hepatic gene’s transcriptional level between the two groups, we performed transcriptome sequencing on total RNA samples from 16 lambs (8 lambs per group). In total, 60.33 ± 2.35 million clean sequence reads were obtained from the liver transcriptome. Among the encoded genes, 245 DEGs were identified from the comparison of the two groups. Among these DEGs, there were 102 upregulated genes and 143 downregulated genes. DEGs in each pair of the two groups of different feeding strategies were functionally annotated by GO analysis. Of the 192 significantly changed GO terms (FDR < 0.05), lipid metabolism that we mainly pay attention to belongs to biological processes, with 54 items, including lipid metabolic process, steroid metabolic process, lipid biosynthetic process, steroid biosynthetic process, cellular lipid metabolic process, lipid catabolic process. As shown in Fig. 7, multiple pathways related to lipid metabolism were identified in the top 20 pathways enriched by KEGG, including steroid hormone biosynthesis, PPAR signaling pathway, steroid biosynthesis, arachidonic acid metabolism, fatty acid metabolism, cholesterol metabolism, fatty acid degradation, biosynthesis of unsaturated fatty acids, glycolysis/gluconeogenesis and propanoate metabolism. We found that *AKR1C1*, *SCD*, *FADS1*, *FADS2*, *CYP7A1*, *CYP4A6*, *ACADM*, *ALDH6A1*, and *ACSS2* were significantly upregulated in the G group; inversely, grazing significantly downregulated *CYP2C23* and *PLB1*. The expression of these 11 genes was validated using qPCR, and the expression trends remained consistent (Fig. 7c).

### Correlation of genes and metabolites in the liver

**Fig. 8:**
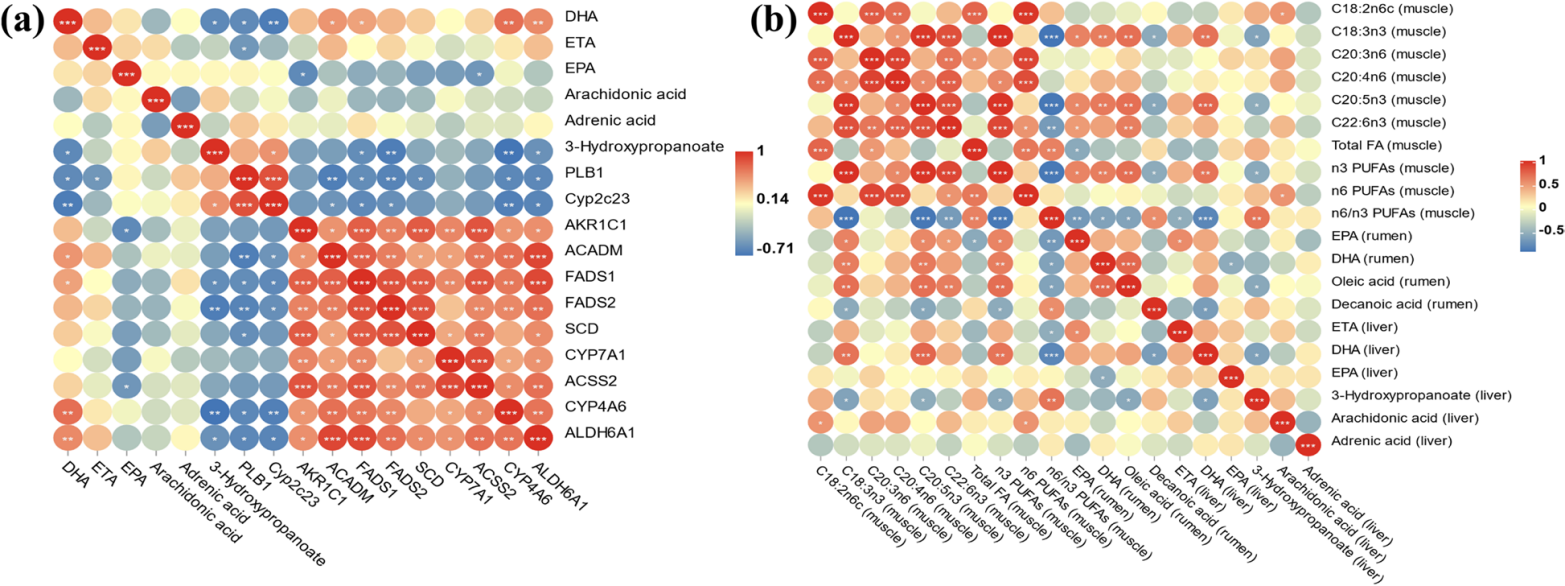
Pearson correlation analysis of lipid-related metabolites and genes in liver (**a**). Pearson correlation analysis of lipid-related metabolites in muscle, rumen and liver (**b**) (*n* = 8 per group). ^*^P < 0.05, ^**^P < 0.01, ^***^P < 0.001. ETA, icosatrienoic acid. DHA, docosahexaenoic acid. EPA, icosapentaenoic acid

On the basis of our biological processes of interest, important metabolites enriched to the unsaturated fatty acid metabolic pathway and the propionate metabolic pathway were subjected to Pearson correlation analysis with differential genes related to lipid metabolism (Fig. 8a). In detail, ETA was negatively correlated with *PLB1*. DHA was found to have a negative correlation with *PLB1* and *CYP2C23*, and a positive correlation with *CYP4A6*, *FADS1*, *ACADM*, and *ALDH6A1*. The 3-hydroxypropanoate was negatively correlated with DHA, *CYP4A6*, *FADS1*, *FADS2*, *ALDH6A1*, and positively correlated with *CYP2C23*.

### Correlations of lipid metabolism in the rumen, liver and muscle

A Pearson correlation analysis was performed to analyze the effect of rumen and liver lipid metabolism on muscle fatty acid deposition (Fig. 8b). We found that EPA and n-3 PUFAs in muscle were significantly positively correlated with EPA, DHA, oleic acid in the rumen and DHA in the liver, and conversely significantly negatively correlated with rumen decanoic acid and hepatic 3-hydroxypropanoate. And there was a significant positive correlation between DHA in muscle and EPA and oleic acid in rumen. In the communication between rumen and liver, EPA in the rumen was positively correlated with ETA in the liver.

## Discussion

For sheep, the rumen and liver are two important organs involved in metabolism and production. Rumen, as a unique and vital digestive organ of ruminants, converts indigestible forage into nutrients, the main energy source of host animals, through its symbiotic microbiota. As the core site of lipid metabolism, the function of liver determines the decomposition, synthesis and deposition of lipids in ruminants. Currently, there is limited knowledge of the difference of rumen microbes and liver metabolism under different feeding patterns. By integrating the rumen metagenome, rumen and liver metabolome and liver transcriptome, we investigated the contribution of rumen microbiome-dependent and host hepatic metabolome-dependent mechanisms to lipid metabolism of the body.

The feeding pattern is a complex system containing various factors, including the level of fiber [22], type of fiber [23], rumen pH [24], etc. Previous researches have determined that various fiber quantities, sources, and pH could change the molar ratio of VFA in the rumen, which then affect the whole FAs metabolism after entering the circulation via the portal vein. Although understanding the variability of these factors is important for a deeper investigation of FAs metabolism, resolving these individual factors separately is difficult to achieve in practical generation. Taken together, it is relevant to analyze the effects on FAs brought about by feeding patterns. The ruminal VFA concentration is the crucial factor that can reflect the impact of different feeding treatments on ruminal fermentation [25]. Similar to a previous study, rumen fermentation characteristics were significantly affected due to the differences in feeding pattern, which represented the increased carbohydrate fermentation in the rumen of indoor feeding lambs [26]. Rumen fermentation is affected by the combination of pH and dietary substrate, and then regulates lipid metabolism. A previous study found that rumen propionate concentration increased with decreasing pH [27], which was consistent with this experiment. In current work, the significantly increased propionate, isovalerate, and valerate in the rumen represented the increased utilization of energy, which indicated more nutrients could be available for growth due to the enhanced rumen energy intake when propionate could be absorbed and converted to glucose, amino acids and lipids [28]. Concisely, these evidences indicated that the change in feeding regime would accordingly alter the rumen fermentation, thereby affecting nutrient absorption and metabolism.

Rumen microbial community structure could be affected by environmental, host, physiological state, and even behavioral characteristics that have evolved together with the varied feeding strategies in ruminants [29]. Recently, a study focused on the gut microbiome of herbivores, suggesting that the richness of the microbiota was increased in animals from the wild environment than in captive animals [30]. In this present study, the richness and evenness of microorganisms were significantly higher in G group than in F group, which was consistent with the results of Xue et al. [26]. A previous paper has shown that the high-grain diet hurts the microbial diversity index of rumen microorganisms [31], which may partly explain this result. In addition, we obtained four unique phyla in G group through metagenomic analysis, which further confirmed that grazing model was more conducive to the diversity of rumen microecology.

The functional differences of microorganisms in bacterial communities were predicted based on Tax4Fun using 16S rRNA sequencing data, and the 3 pathways screened were related to lipid metabolism. These preliminary results indicated that there were differences in lipid metabolism between the two groups of microorganisms. The key differential microorganisms *Butyrivibrio_sp_AC2005* and Acidaminococcales, both screened by LEfSe analysis, were found to be closely associated with lipid metabolism, providing further confirmation of the above speculation [32, 33]. Interestingly, *Succiniclasticum*, as a core component of the differential rumen microbiome, has the ability to convert succinate to propionate [34, 35], hence its enhancement in the F group could be a logical explanation for the augment in the propionate in the rumen liquid of the stall-feeding lambs. Besides, the significant positive correlation of *Succiniclasticum* abundance with ruminal propionate concentration further corroborated our point. In addition, although there is no direct evidence, some studies have found that the synergistic reduction of *Coprobacillus* and rumen succinate improved dyslipidemia caused by a high-fat diet, and *Candidatus_Saccharimonas* was positively correlated with the proportion of propionate in rumen [35, 36]. Both suggested that *Coprobacillus* and *Candidatus_Saccharimonas* were directly or indirectly involved in the rumen production of succinate and propionate, respectively, and further confirmed the vital function of propionate in lipid metabolism between the two groups. Meanwhile, it was obvious that the dietary composition of lambs under the two feeding modes was significantly different, especially in terms of carbohydrates, due to the concentrate supplement in the F group. So, we used the CAZyme database for comparison, and found that the abundance of typical endoglucanase GH45 and four GTs family enzymes genes were higher in the F group. GHs cleave bonds by the insertion of a water molecule to hydrolyze complex carbohydrates, while GTs assemble complex carbohydrates from activated sugar donors [37, 38]. A recent study reported that the increased abundance of GTs gene in rumen of dairy cows promoted the production of volatile acids, which further promoted the production activities [39]. Similarly, compared to the G group, the stall-feeding lambs had higher abundances of genes encoding CAZymes involved in carbohydrate degradation (GH45) and synthesis (GTs), as well as higher concentration of major VFAs in the rumen, indicating that the rumen microbiomes of F group might be more efficient to generate VFAs, and therefore provide more energy for growth in host sheep.

After entering the rumen, dietary lipids are hydrolyzed to release FAs, which are metabolized by rumen microorganisms, including degradation, synthesis and hydrogenation. Nevertheless, rumen microbial degradation of FAs is less than 1% of the total fatty acid [40]. Moreover, the synthesis of FAs by rumen microorganisms is also very small, because rumen microbes prefer to directly utilize dietary FAs rather than synthesize them themselves [41]. Therefore, due to the above characteristics of the metabolism of FAs by rumen microorganisms, the hydrogenation of rumen microorganisms is the main factor that ultimately affects FAs composition in ruminant products. The effects of different FAs on human health are diverse, and SFAs can increase the risk of cardiovascular disease. The FAs that have positive effects on human health are mainly UFAs, especially PUFAs, which play a role in regulating cell cycle, reducing body fat, and preventing cardiovascular diseases. However, the biohydrogenation of PUFAs in rumen will generate a large number of SFAs, which will reduce the deposition of PUFAs in ruminant products [42]. According to rumen metabolome analysis, EPA, DHA and oleic acid were down-regulated and decanoic acid was up-regulated in the F group. Moreover, Tenericutes, which significantly increased in abundance under stall-feeding condition, was found to have a role in biohydrogenation to convert PUFA to SFA [43]. Therefore, it could be speculated that indoor feeding to some extent enhanced the ruminal biohydrogenation by increasing Tenericutes, leading to the accumulation of SFA. It has been found that biohydrogenation of PUFAs in the rumen was lower in hay-fed animals than that in concentrate-fed animals, with a greater percentage of PUFAs bypassing the rumen in the former than that in concentrate-fed animals [44]. Based on the sensitivity of rumen microorganisms to diets, this phenomenon may be caused by the difference in diet components. For PUFAs, the balance of n-3 PUFAs (including EPA and DHA) and n-6 PUFAs is an important index used to evaluate the nutritional value of meat quality for humans [45]. Previous studies of our team have found that grazing model increased the content of n-3 PUFAs and n-6 PUFAs and reduced the ratio of n-6/n-3 in LD muscle, which is more beneficial to human health [6]. The substantial positive association between EPA and DHA in rumen and EPA and n-3 PUFAs content in muscle indicated that the rumen of grazing lambs produced more EPA and DHA and thus potentially up-regulated the n-3 PUFAs content in muscle, contributing to better lamb meat quality. Besides, the accumulation of n-3 PUFAs could inhibit lipid synthesis in liver and reduce cholesterol, triglycerides, and LDL in plasma [46]. These results suggested that the decrease of LDL in plasma and triglyceride, cholesterol, FAS and ACC in liver by grazing may be related to the up-regulation of n-3 PUFAs, leading to the decrease of lipid metabolic activity in the liver.

In ruminants, some UFAs are hydrogenated in the rumen and further metabolized in various tissues, including the liver, with important roles in lipid and lipoprotein metabolism. Therefore, manipulation of muscle fatty acid composition should take into account liver metabolism, especially in the biosynthesis of n-3 PUFAs [47]. Through liver metabolomics, we found that ETA enriched in the biosynthesis of unsaturated fatty acids pathway was up-regulated in the G group, which belongs to n-3 PUFAs. During the synthesis of long-chain PUFAs, ETA can be desaturated by Δ5-desaturase to produce EPA, which provides a precursor for more deposition of EPA in muscle [48]. In the liver, the mechanism of formation of 3-hydroxypropanoate involves the conversion of propionate to propionyl-CoA, which is reduced to acrylyl-CoA, followed by hydration of acrylyl-CoA to 3-hydroxypropanoate-CoA, and hydrolysis to 3-hydroxypropanoate [49]. Combined with the positive correlation between ruminal propionate and the biomarker 3-hydroxypropanoate in the liver in Table S4, it followed that the increased ruminal propionate was absorbed by the liver, resulting in changes in hepatic propionate metabolism in the F group. At the same time, the citric acid cycle would also be regulated by the metabolism of propionate in the liver. It has been reported that the increase of citric acid stimulated adipogenesis and gluconeogenesis by activating ACC, which was compatible with the results of increased ACC in liver enzyme activity in the F group [50]. Meanwhile, due to gluconeogenesis in liver, propionate can indirectly modulate adipose tissue lipogenesis through increased glucose availability [47]. Furthermore, the role of propionate in reducing the synthesis of long-chain PUFAs in the liver has also been previously found [51]. Therefore, the negative correlation between hepatic 3-hydroxypropanoate and n-3 PUFAs in the muscle suggested that the downregulation of muscle PUFAs in the F group may be related to the changes in propionate metabolism.

Based on the concerning biology processes, we focused on the expression profile of eleven lipid metabolic-related genes involved in the signal pathway of liver. Among them, *AKR1C1*, *SCD*, *FADS1*, *FADS2*, *CYP7A1*, *CYP4A6*, *ACADM*, *ALDH6A1* and *ACSS2* were significantly up-regulated in the G group; inversely, grazing significantly down-regulated *CYP2C23* and *PLB1* which were essential factors for the variations in liver lipid metabolism under different feeding patterns. Further correlation analysis revealed the major roles of *FADS1*, *FADS2*, *CYP4A6*, *ACADM*, *ALDH6A1*, *PLB1*, and *CYP2C23* in the metabolism of UFAs. *FADS1* and *FADS2*, encode enzymes involved in the conversion of α-linolenic acid to EPA, DPA, and DHA and linoleic acid to γ-linolenic acid and arachidonic acid, respectively [52]. A study in bovine mammary epithelial cells found a significant positive correlation between the contents of EPA and the expression of *FADS1* [53]. Another previous study has reported that reduced activity of the desaturase enzymes mediated by *FADS1* and *FADS2* leads to a reduction of PUFAs in plasma [54]. Combined with the significant positive correlation between DHA and *FADS1*, it was speculated that PUFAs deposition in the G group was related to the up-regulation of *FADS1* gene. It is well known that meat quality is strongly related to fatty acid composition, especially the proportion of PUFAs. Our previous results found that while indoor feeding increased daily gain, it enhanced the proportion of n-6/n-3 PUFAs, which negatively affected meat quality [6]. Similarly, it has been reported that the transcription of *FADS2* could affect the endogenous transformation of long-chain PUFAs, reducing the bioavailability of n-3 PUFAs and promoting the accumulation of n-6 PUFAs [55]. Though the exact mechanism is not clear, it further confirms the important role of *FADS2* gene in the conversion of long-chain PUFAs. CYP4A6 is a member of the cytochrome P450 IVA gene subfamily, which encodes several enzymes that catalyze the metabolic process of SFAs and UFAs, including arachidonic acid, and plays an essential role in the metabolism of FAs [56]. Medium-chain acyl-CoA dehydrogenase, encoded by the *ACADM* gene, catalyzes the first step of β-oxidation. Previous studies showed that *ACADM* gene knockdown remarkably enhanced lipid accumulation in vitro, implying that the decrease of triglyceride and cholesterol in liver of grazing group may be related to *ACADM* upregulation [57]. According to a study, ALDH6A1 was identified as a new adipose tissue marker associated with obese people through its involvement in propionate metabolism, further suggesting a major function for propionate in lipid metabolism [58]. A series of n-3 PUFAs such as EPA can act as an efficient substrate of CYP2C23 enzyme, so downregulation of the *CYP2C23* gene may lead to the accumulation of n-3 PUFAs in the liver [59]. The significant negative correlation between *CYP2C23* and DHA also supports this conjecture. PLB1 is a secreted enzyme with lysophospholipase hydrolase and lysophospholipase transacylase activities, which is required for the release of arachidonic acid from phospholipids [60]. EPA can be synthesized by both ETA and arachidonic acid pathways, respectively [48]. Owing to the negative relationship between *PLB1* and ETA, it was hypothesized that the deletion of the arachidonic acid pathway caused by the down-regulation of *PLB1* might be compensated by the up-regulation of ETA, which finally did not affect the synthesis of EPA in the liver.

Notably, correlation analysis also revealed that 3-hydroxypropanoate, the important DFMs in the propionate metabolism pathway were significantly correlated with *CYP4A6*, *FADS1*, *FADS2*, *ALDH6A1* and *CYP2C23*. Previous reports on the influence of propionate metabolism on liver lipid metabolism, suggested that the above changes of lipid metabolic-related genes may be mediated by propionate metabolism [61, 62]. Combined with the result in Table S4 that ruminal propionate content had a significant positive correlation with 3-hydroxypropanoate in the liver, it further demonstrated that microbial-driven propionate production may mediate changes in hepatic propionate metabolism and subsequently participate in the regulation of multiple fatty acid metabolism-related signaling pathways. Moreover, based on the close correlation between UFAs in muscle and liver, it is implied that changes in hepatic lipid metabolism potentially may lead to differences in fatty acid deposition in muscle.

## Conclutions

In summary, our findings addressed the variations in lipid metabolism between grazing and stall-feeding lambs, from the rumen to the liver. By reducing the abundance of *Succiniclasticum*, the grazing pattern decreased the propionate content and changed the propionate metabolism of rumen. The rumen wall absorbed more propionate from stall-feeding sheep, which reached the liver and changed the propionate metabolism and citrate cycle in the liver. Meanwhile, 3-hydroxypropanoate, the key DFM enriched in the propionate metabolism pathway, was significantly correlated with lipid-related genes *CYP4A6*, *FADS1*, *FADS2*, *ALDH6A1*, and *CYP2C23*, suggesting that propionate metabolism may regulate hepatic lipid metabolism. In addition, the decreased abundance of Tenericutes in grazing sheep weakened the hydrogenation of UFAs, leading to the accumulation of EPA, DHA and oleic acid and the reduction of decanoic acid in rumen, which also became a potential reason for the up-regulation of UFAs in muscle. Overall, microbial-mediated metabolic changes in the rumen of grazing sheep were important in affecting ruminal and hepatic lipid metabolism, and may further contribute to the deposition of FAs in muscle, but the specific mechanisms in meat need to be more explored.

## Supplementary Information

**Additional file 1: Table S1.** Composition and nutrient level of the experimental diet. **Table S2.** Good's coverage of rumen microbial diversity of sheep under different feeding regimes. **Table S3.** Pearson correlation analysis between microorganisms and VFA in rumen. **Table S4.** Pearson correlation analysis between ruminal VFAs and lipid-related metabolites in liver. **Fig. S1.** Venn diagram illustrating the overlap of microbial phyla between the two groups in metagenomic sequencing (**a**). Comparisons of the pathway annotation based on the KEGG database of rumen microbiomes in the two groups by the Welch's *t*-test (**b**). Metabolic pathway enrichment score in metagenomic sequencing (**c**). **Fig. S2.** Score plot of discriminant analysis of squares (PLS-DA) model obtained in positive (**a**) and negative mode (**b**) of rumen metabolism. Score plot of PLS-DA model obtained in positive (**c**) and negative mode (**d**) of liver metabolism. **Fig. S3.** Functional enrichment analysis of differential metabolites in rumen (**a**) and liver (**b**).

## Data Availability

The datasets used and analysed during the current study are available from the corresponding author on reasonable request.

## Abbreviations

ACC: Acetyl-CoA carboxylase
BUN: Blood urea nitrogen
DEGs: Differential expression genes:
DFMs: Differential metabolites
DHA: Docosahexaenoic acid
EPA: Icosapentaenoic acid
ETA: Icosatrienoic acid
FAS: Fatty acid synthase
FAs: Fatty acids
HDL: High-density lipoprotein
LD: Longissimus dorsi
LDL: Low-density lipoprotein
NEFA: Nonestesterified fatty acid
PUFAs: Polyunsaturated fatty acids
SFAs: Saturated fatty acids
T-AOC: Total antioxidant capacity
UFAs: Unsaturated fatty acids
VFA: Volatile fatty acid
