# Supplementary material for: Rumen microbial-driven metabolite from grazing lambs potentially regulates body fatty acid metabolism by lipid-related genes in liver
Source: J Anim Sci Biotechnol. 2023 Mar 7;14:39. doi: 10.1186/s40104-022-00823-y (PMC9990365; doi:10.1186/s40104-022-00823-y)
Supplement: Supplementary file 1 — Additional file 1: Table S1. Composition and nutrient level of the experimental diet. Table S2. Good's coverage of rumen microbial diversity of sheep under different feeding regimes. Table S3. Pearson correlation analysis between microorganisms and VFA in rumen. Table S4. Pearson correlation analysis between ruminal VFAs and lipid-related metabolites in liver. Fig. S1. Venn diagram illustrating the overlap of microbial phyla between the two groups in metagenomic sequencing (a). Comparisons of the pathway annotation based on the KEGG database of rumen microbiomes in the two groups by the Welch's t-test (b). Metabolic pathway enrichment score in metagenomic sequencing (c). Fig. S2. Score plot of discriminant analysis of squares (PLS-DA) model obtained in positive (a) and negative mode (b) of rumen metabolism. Score plot of PLS-DA model obtained in positive (c) and negative mode (d) of liver metabolism. Fig. S3. Functional enrichment analysis of differential metabolites in rumen (a) and liver (b). [file 40104_2022_823_MOESM1_ESM.docx]

**Supplementary Information**

**Rumen microbial-driven metabolite from grazing lambs potentially regulates body fatty acid metabolism by lipid-related genes in liver**

Zhen Li, Xingang Zhao, Luyang Jian, Bing Wang, Hailing Luo^*^

State Key Laboratory of Animal Nutrition, College of Animal Science and Technology, China Agricultural University, Beijing 100193, P. R. China

^*^Corresponding author: luohailing@cau.edu.cn

Zhen Li: lizhen6394@126.com

Xingang Zhao: 1404010216@cau.edu.cn

Luyang Jian: jianluyang@cau.edu.cn

Bing Wang: wangb@cau.edu.cn

Hailing Luo: luohailing@cau.edu.cn

**Additional file 1**

**Table S1** Composition and nutrient level of the experimental diet (dry-matter basis)

| **Ingredients** | **Content, %** | **Components** | **Content, %** |
| --- | --- | --- | --- |
| Pellets |  |  |  |
| Corn | 25.24 | Dry matter | 93.17 |
| Alfalfa powder | 25.00 | Crude protein | 13.96 |
| Wheat bran | 7.76 | Crude fiber | 22.74 |
| Rapeseed cake | 6.00 | Ether extract | 3.32 |
| Soybean meal | 8.00 | Crude ash | 9.55 |
| NaHCO_3_ | 1.00 | Calcium | 0.60 |
| Salt | 1.00 | Phosphorus | 0.35 |
| Premix^1^ | 1.00 | Metabolizable energy, MJ/kg^2^ | 9.39 |
| Hay |  |  |  |
| Alfalfa hay  Corn straw  Total | 10.00 15.00 100.00 |  |  |

^1^ Each 1 kg premix contained 320,000 IU vitamin A, 10,000 IU vitamin E, 300 mg copper, 5,000 mg iron, 5,000 mg zinc, 1,000 mg manganese, 40 mg iodine, 10 mg cobalt, and 10 mg selenium

^2^ The metabolizable energy was calculated value

**Table S2** Good's coverage of rumen microbial diversity of sheep under different feeding regimes

| **Simple** | **Good’s_coverage** |
| --- | --- |
| F-2 | 0.9998 |
| F-3 | 0.99986 |
| F-4 | 0.999778 |
| F-5 | 0.999856 |
| F-6 | 0.999834 |
| F-7 | 0.999748 |
| F-8 | 0.999936 |
| G-1 | 0.999829 |
| G-2 | 0.999819 |
| G-3 | 0.999848 |
| G-4 | 0.99991 |
| G-5 | 0.999834 |
| G-6 | 0.999908 |
| G-7 | 0.99979 |
| G-8 | 0.99976 |

“F” means indoor feeding group; “G” means grazing group

**Table S3** Results of Pearson correlation analysis between microorganisms and VFA in rumen^1,2,3^

| **Items** | | **Acetate** | **Propionate** | **Isobutyrate** | **Butyrate** | **Isovalerate** | **Valerate** | **Acetate/**  **propionate** | **Total VFA** |
| --- | --- | --- | --- | --- | --- | --- | --- | --- | --- |
| Tenericutes | *r* | 0.345 | 0.475 | 0.264 | 0.374 | 0.088 | 0.688 | -0.241 | 0.544 |
|  | *P* | 0.655 | 0.525 | 0.736 | 0.626 | 0.912 | 0.312 | 0.565 | 0.163 |
| *Succiniclasticum* | *r* | 0.287 | 0.973^*^ | 0.170 | 0.042 | 0.503 | 0.647 | -0.515 | 0.762^*^ |
|  | *P* | 0.713 | 0.027 | 0.830 | 0.958 | 0.497 | 0.353 | 0.192 | 0.028 |
| *Butyrivibrio_sp_AC2005* | *r* | -0.821 | -0.295 | -0.790 | -0.866 | -0.567 | -0.842 | 0.191 | -0.300 |
|  | *P* | 0.179 | 0.705 | 0.210 | 0.134 | 0.433 | 0.158 | 0.651 | 0.471 |
| Acidaminococcales | *r* | 0.289 | 0.974^*^ | 0.172 | 0.045 | 0.504 | 0.650 | -0.522 | 0.754^*^ |
|  | *P* | 0.711 | 0.026 | 0.828 | 0.955 | 0.496 | 0.350 | 0.184 | 0.031 |
| *Candidatus_Saccharimonas* | *r* | 0.648 | 0.762 | 0.549 | 0.568 | 0.531 | 0.950^*^ | -0.540 | 0.752^*^ |
|  | *P* | 0.352 | 0.238 | 0.451 | 0.432 | 0.469 | 0.050 | 0.167 | 0.031 |
| *Coprobacillus* | *r* | 0.308 | 0.681 | 0.201 | 0.269 | 0.137 | 0.740 | -0.504 | 0.284 |
|  | *P* | 0.692 | 0.319 | 0.799 | 0.731 | 0.863 | 0.260 | 0.203 | 0.495 |

^1^ *r*: correlation coefficient; *P*: significance (probability)

^2^ Significant correlation was considered at *P* < 0.05 and marked with “*”

^3^ *VFA* volatile fatty acid

**Table S4** Results of Pearson correlation analysis between ruminal VFAs and lipid-related metabolites in liver^1,2^

| **Items^3^** |  | **Acetate** | **Propionate** | **Isobutyrate** | **Butyrate** | **Isovalerate** | **Valerate** |
| --- | --- | --- | --- | --- | --- | --- | --- |
| ETA | *r* | -0.142 | -0.192 | 0.077 | -0.054 | 0.025 | -0.284 |
|  | *P* | 0.660 | 0.551 | 0.811 | 0.867 | 0.940 | 0.371 |
| EPA | *r* | 0.613^*^ | 0.698^*^ | 0.349 | 0.459 | 0.593^*^ | 0.691^*^ |
|  | *P* | 0.034 | 0.012 | 0.266 | 0.133 | 0.042 | 0.013 |
| DHA | *r* | -0.637^*^ | -0.573 | -0.215 | -0.661^*^ | -0.281 | -0.607^*^ |
|  | *P* | 0.026 | 0.051 | 0.502 | 0.019 | 0.377 | 0.036 |
| 3-hydroxypropanoate | *r* | 0.633^*^ | 0.617^*^ | 0.646^*^ | 0.751^**^ | 0.728^**^ | 0.724^**^ |
|  | *P* | 0.027 | 0.033 | 0.023 | 0.005 | 0.007 | 0.008 |

^1^ *r*: correlation coefficient; *P*: significance (probability)

^2^ Significant correlation was considered at *P* < 0.05 and marked with “*”

^3^ *ETA* Icosatrienoic acid, *DHA* Docosahexaenoic acid, *EPA* Icosapentaenoic acid


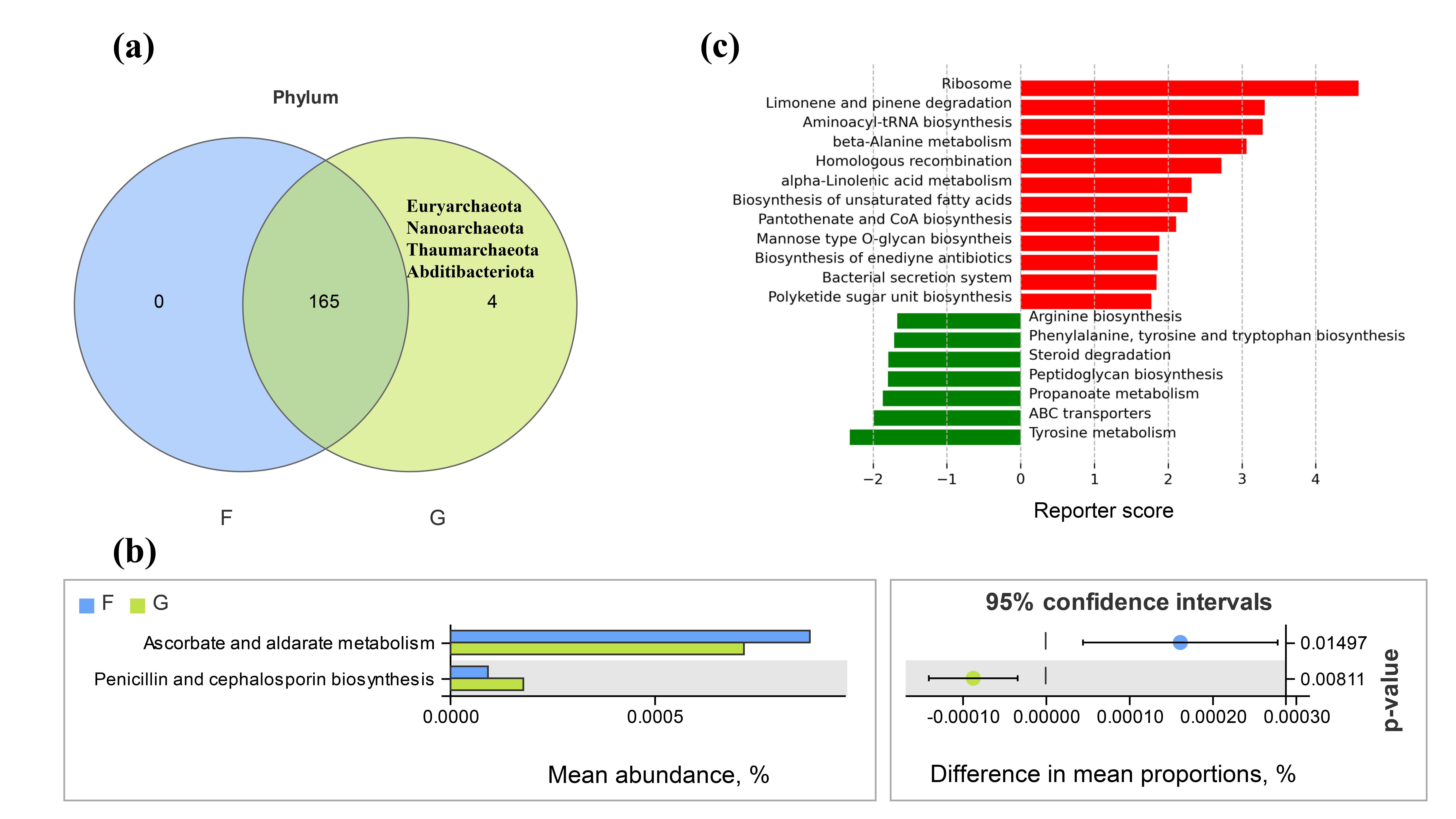


**Fig. S1** Venn diagram illustrating the overlap of microbial phyla between the two groups in metagenomic sequencing (**a**). Comparisons of the pathway annotation based on the KEGG database of rumen microbiomes in the F and G groups by the Welch's *t*-test (**b**). Metabolic pathway enrichment score in metagenomic sequencing (**c**). Positive/negative values indicate a significant increase/decrease of abundance in the grazing group, respectively, and larger absolute values indicate more significant enrichment


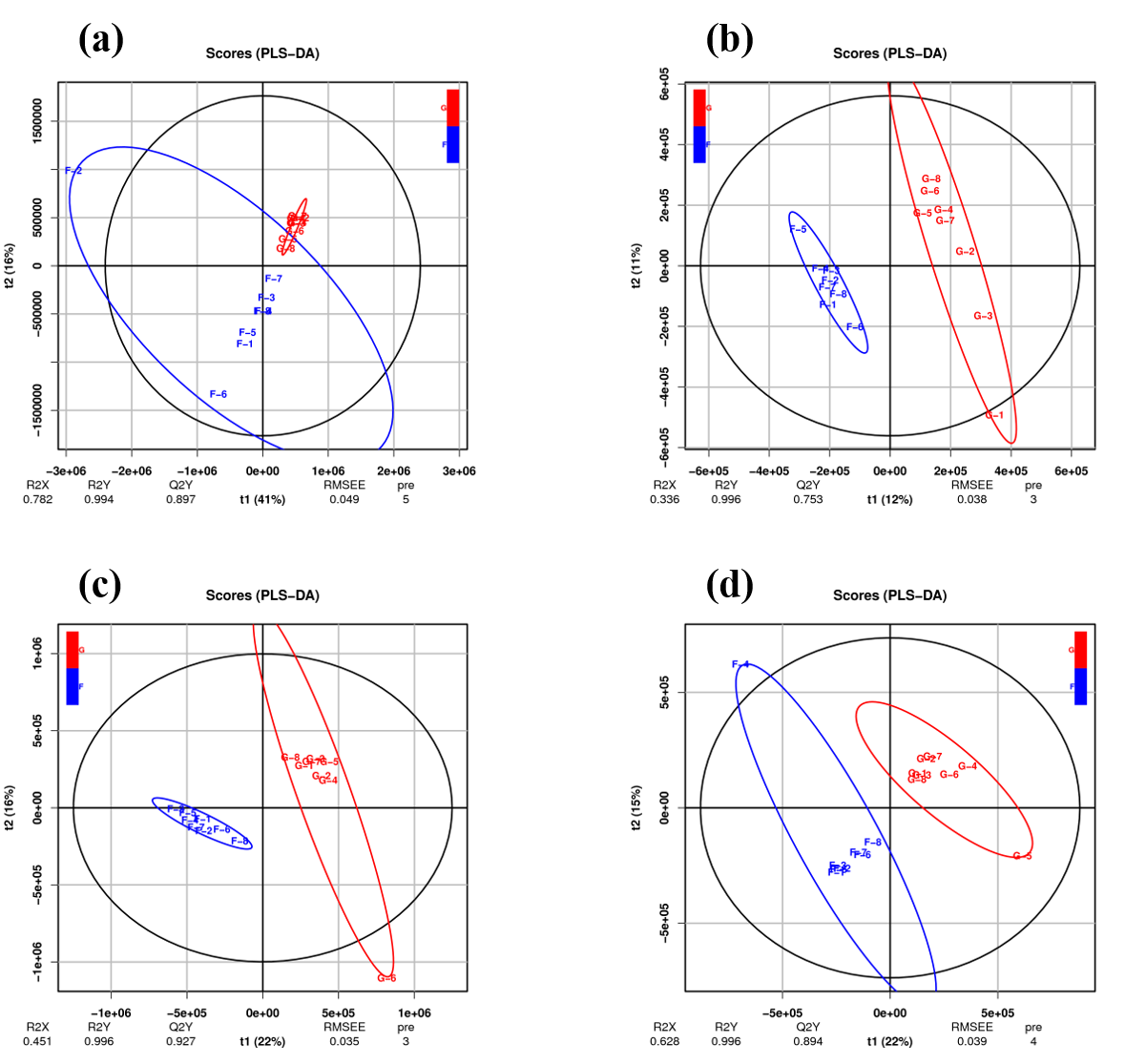


**Fig. S2** Score plot of partial least squares discriminant analysis (PLS-DA) model obtained in positive (**a**) and negative mode (**b**) of rumen metabolism. Score plot of PLS-DA model obtained in positive (**c**) and negative mode (**d**) of liver metabolism





**Fig. S3** Functional enrichment analysis of differential metabolites in rumen (**a**) and liver (**b**)
